# Chemical genetic inhibition of DEAD-box proteins using covalent complementarity

**DOI:** 10.1093/nar/gky706

**Published:** 2018-08-08

**Authors:** Krister J Barkovich, Megan K Moore, Qi Hu, Kevan M Shokat

**Affiliations:** 1 Department of Cellular and Molecular Pharmacology, University of California, San Francisco, CA 94158, USA; 2 Howard Hughes Medical Institute, University of California, San Francisco, CA 94158, USA; 3 Department of Chemistry, University of California, Berkeley, CA 94720, USA

## Abstract

DEAD-box proteins are an essential class of enzymes involved in all stages of RNA metabolism. The study of DEAD-box proteins is challenging in a native setting since they are structurally similar, often essential and display dosage sensitivity. Pharmacological inhibition would be an ideal tool to probe the function of these enzymes. In this work, we describe a chemical genetic strategy for the specific inactivation of individual DEAD-box proteins with small molecule inhibitors using covalent complementarity. We identify a residue of low conservation within the P-loop of the nucleotide-binding site of DEAD-box proteins and show that it can be mutated to cysteine without a substantial loss of enzyme function to generate electrophile-sensitive mutants. We then present a series of small molecules that rapidly and specifically bind and inhibit electrophile-sensitive DEAD-box proteins with high selectivity over the wild-type enzyme. Thus, this approach can be used to systematically generate small molecule-sensitive alleles of DEAD-box proteins, allowing for pharmacological inhibition and functional characterization of members of this enzyme family.

## INTRODUCTION

Small molecule inhibitors are powerful tools for the study of cellular enzymatic processes due to their rapid onset of inhibition, which prevents cellular compensation and their ability to be administered at varying doses, allowing for partial as well as complete loss-of-function phenotypes. As compared to the adenosine triphosphate (ATP)-binding site of kinases, the development of small molecules targeting the nucleotide-binding pocket of adenosine triphosphatases (ATPases) has been proven challenging. ATP-competitive inhibitors of the AAA+ ATPase p97/VCP and structurally related family members have been discovered (1,2), although a generalizable small molecule scaffold with high affinity for the ATPase nucleotide-binding pocket has not yet been identified. This is likely due to the reliance on electrostatic interactions for high-affinity binding with its native substrate (ATP). Even if a suitable uncharged pharmacophore of the tri- or diphosphate could be identified, the high conservation of this site across >400 human proteins would make identifying a selective inhibitor of a single member of the family a significant challenge (3,4). As such, it is difficult to develop potent small molecule inhibitors of most ATPases, including the DEAD-box proteins.

DEAD-box proteins are the largest family of enzymatic RNA chaperones in humans (5). Named for their conserved Walker B motif consisting of adjacent aspartate-glutamate-alanine-aspartate (D-E-A-D) residues, DEAD-box proteins are required for all stages of RNA metabolism including transcription, processing and splicing, export, translation and decay (6–8). DEAD-box proteins bind nucleotides via the canonical Walker A and B motifs and the family-specific Q-motif that recognizes the adenine of ATP and makes the DEAD-box proteins ATP-specific (3,4,9). ATP binding and hydrolysis drive non-processive unwinding of RNA substrates by local strand separation (10,11). Yet despite the successful biochemical and structural characterization of this essential family of enzymes, our understanding of the specific RNA substrates acted upon by DEAD-box proteins remains poorly understood (6).

Owing to their roles in essential cellular processes, DEAD-box proteins are often misregulated in human disease and have been identified as potential pharmaceutical targets in cancer and viral and bacterial infections (12,13). However, specific chemical targeting of a single member of the DEAD-box family is challenging. Several natural product inhibitors of eIF4A have been identified, including hippuristanol and silvestrol (14,15), and Takeda Pharmaceuticals recently published synthetic small molecules targeting eIF4AIII and Brr2 (16,17). However, these compounds all rely on targeting cryptic allosteric pockets for their specific inhibition and as such they are highly selective yet are unlikely to be good structural starting points for discovery of inhibitors for other members of the DEAD-box family.

Although genetic and biochemical methods have been invaluable in the advancement of our understanding of DEAD-box proteins, they are fundamentally limited. Genetic knockout and loss of function mutants require extensive selection and verification (18,19) during which time cellular compensation may obscure the primary role of the protein being studied. DEAD-box proteins are also often essential, further complicating these loss-of-function studies (6). Gene knockout studies of structurally similar enzymes such as DEAD-box proteins may additionally be subject to compensation by partially redundant family members (20,21). The use of temperature-sensitive mutants in *Saccharomyces cerevisiae* partially solves these problems (22), although temperature-sensitive mutant enzyme inactivation often occurs through poorly understood mechanisms and temperature changes may alter temperature-sensitive processes such as RNA homeostasis. Pharmacologically controllable fusion proteins would be a potential avenue to acutely regulate DEAD-box protein function (23), although the multiprotein complexes in which these proteins function may complicate fusion protein design.

A potential solution to these challenges is to utilize the tools of chemical genetics. Previous chemical genetic approaches achieved specificity to the ATPases myosin-1β and kinesin through analog-sensitive (AS) alleles generated by space-creating mutations adjacent to the N6-position of ATP (24,25). As further evidence for the importance of charged small molecules for targeting the ATPase nucleotide-binding pocket, both studies developed nucleotide di- and triphosphate-based inhibitors and thus were limited by the inherent affinity of nucleotides for this pocket. This class of small molecules is additionally challenging to modify to develop cell-active compounds (26). Recently, we reported an effort to identify AS mutants of the DEAD-box protein DDX3 (27). We identified a space creating mutation generated through mutation of a conserved aromatic residue in the adenine-binding pocket, which showed a 100-fold reduction in biochemical activity. Yeast expressing this mutant in DDX3-homolog Ded1 displayed a temperature-sensitive phenotype, suggesting that increasing the size of the ATP pocket leads to a hypomorphic allele. We therefore turned to another strategy for imparting drug sensitivity into the ATP-binding pocket of DEAD-box proteins.

**Figure 1.**
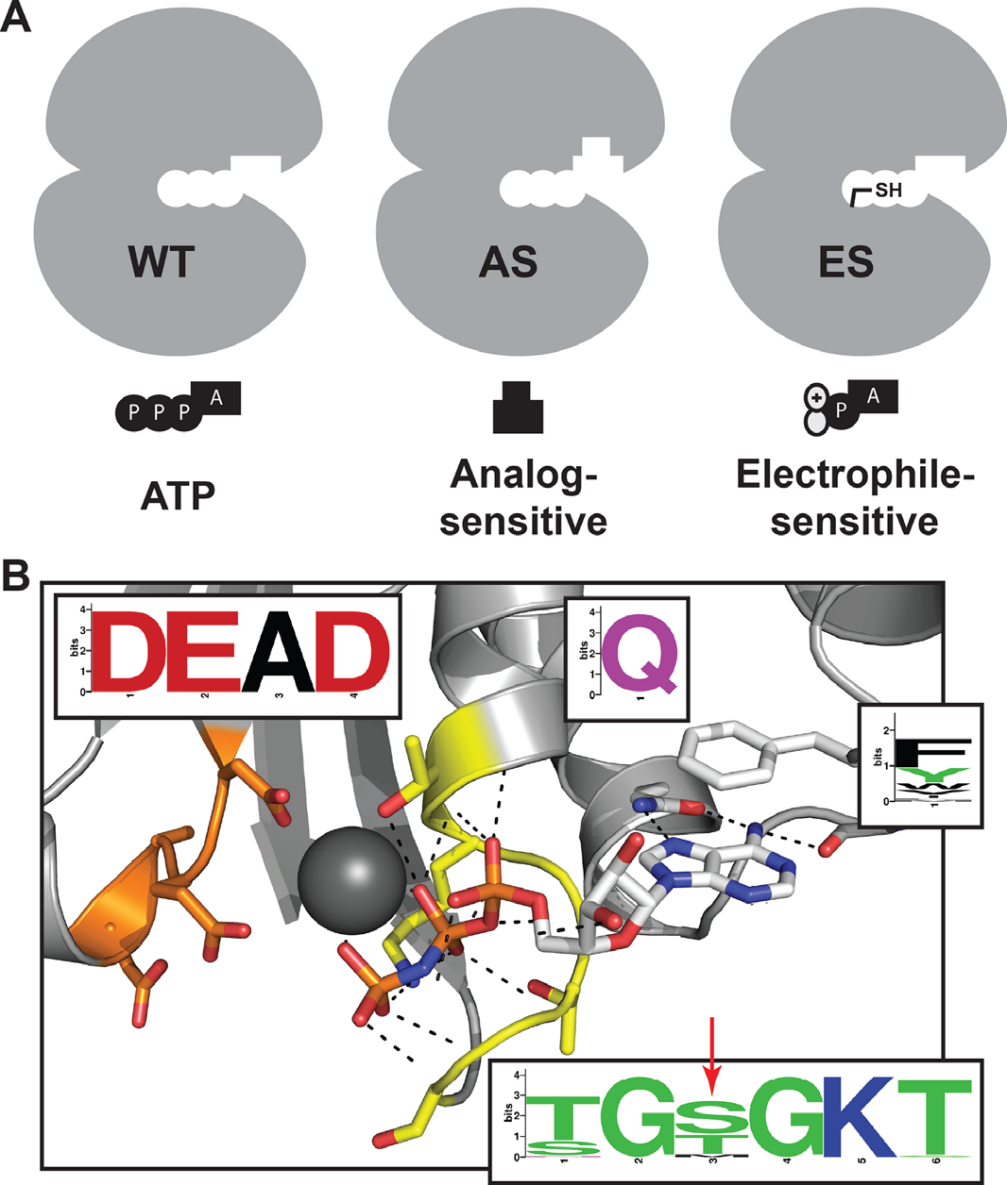
Development of an ES mutation in DEAD-box proteins. (**A**) AS chemical genetic strategies rely on space-creating mutations and bulky inhibitors, while ES strategies rely on cysteine-electrophile covalent complementarity. (**B**) Conserved nucleotide-binding site of DEAD-box proteins including the P-loop/Walker A (yellow), DEAD-box/Walker B (orange) and Q-motif (white) with conservation across all human DEAD-box proteins (inserts) and the site of ES mutation identified (red arrow; PDB ID: 2HXY).

Cysteine is the most nucleophilic of the 20 natural amino acids and is the second least common amino acid, after tryptophan. Additionally, cysteine-reactive small molecules are clinically approved and stable *in vivo* (28). These properties provide an ideal scenario for introduction of a cysteine into the binding pocket of a DEAD-box protein and targeting it for irreversible inhibition. Non-native cysteines have previously allowed for specific chemical inhibition of challenging targets (29) and kinases expressing non-endogenous cysteines have been targeted in an ‘electrophile-sensitive’ (ES) chemical genetic approach (30–32), analogous to the ‘AS’ approach (Figure 1A). This approach may be especially beneficial in DEAD-box proteins because of the difficulty in generating space-creating mutations in the ATPase active site that do not adversely affect enzyme function (27). In fact, a previous study found that sensitivity to the semi-selective cysteine-targeting small molecule N-ethylmaleimide could be transferred to other ATPases through mutation of an active-site residue to cysteine (33,34).

In this work, we describe a method for the inhibition of specific DEAD-box proteins using covalent complementarity. We identify a site of low conservation in the P-loop of DEAD-box proteins that can be mutated to cysteine without a substantial reduction in enzyme activity. We then develop a series of electrophile-containing small molecules that target this cysteine and specifically inhibit ES, but not wild-type DEAD-box proteins. Analysis of the drug bound complex in DDX3 reveals that the formation of a covalent bond between the engineered cysteine and the electrophilic inhibitor retains the adenine base interactions but requires a reorientation of the P-loop. Taken together, these results demonstrate that chemical genetic inhibition of the DEAD-box protein family is possible through targeting the ATP-binding site and identify novel small molecules for the biochemical inhibition of these enzymes.

## MATERIALS AND METHODS

### Multiple sequence alignment

Sequences for all human DEAD-box proteins were obtained from NCBI. All visualizations were generated by WebLogo (weblogo.berkeley.edu) (35).

### Recombinant protein purification

DDX3 (132–406), DDX3 (132–607) and v-Src were expressed and purified as previously described (27,36). Full-length Ded1 and Dbp2 were expressed as 6xHis–SUMO fusion proteins in *Escherichia coli* BL21 (DE3) cells. RIG-I (230–795) was expressed as a 6xHis–MBP fusion protein. Cultures were grown to OD ∼0.8 then protein expression was induced with 0.5 mM isopropyl β-D-1-thiogalactopyranoside (IPTG) at 16°C overnight. Cells were lysed by microfluidizer, clarified at 20 000 × g for 25 min and purified by nickel chromatography including a 1 M NaCl wash to remove bound nucleic acids. Eluted protein was incubated with SUMO protease and dialyzed into 25 mM Tris (pH 7.5), 300 mM NaCl and 0.5 mM tris(2-carboxyethyl)phosphine (TCEP) overnight at 4°C. The sample was then purified by heparin chromatography and eluted at ∼700 mM NaCl, 25 mM Tris (pH 7.5) and 0.5 mM TCEP. Fractions containing pure protein, as analyzed by Coomassie staining, were concentrated to at least 50 μM, supplemented with 10% glycerol and snap frozen in liquid nitrogen.

### Coupled ATPase assays

Assays were performed as previously described (27). All experimental results are reported as the average of three replicates with error bars representing the standard deviation of the results.

### RNA duplex unwinding assays

RNA duplex unwinding assays were performed as previously described (37) using 1 nM duplex RNA, 2 mM ATP/MgCl_2_ and 1 μM DDX3 (132–607), 0.5 μM Ded1 or 0.5 μM Dbp2. Reaction buffer contained 20 mM 4-(2-hydroxyethyl)piperazine-1-ethanesulfonic acid (HEPES) (pH 7.5), 100 mM NaCl, 0.5 mM MgCl_2_, 1 mM TCEP, 0.01% (v/v) octylphenoxypolyethoxylethanol (NP-40) and 5% glycerol. The sequences of the RNA duplex strands are 5′-GCUUUACGGUGC-3′ and 5′-GAACAACAACAACAACCAUGGCACCGUAAAGC-3′. For experiments that included chemical inhibitors, RNA, protein and inhibitors were pre-incubated in reaction buffer for 5 min prior to addition of ATP-Mg. Results were quantified using ImageJ (NIH) (38). All experimental results are reported as the average of three replicates with error bars representing the standard error of the results.

### Yeast genetics

For a complete list of yeast strains and plasmids used in this work, see Supplementary Data. A strain of *S. cerevisiae* containing a deletion of the DED1 locus complemented by URA3-marked plasmid containing DED1 was previously described (39). HA-eIF4A^ES^*S. cerevisiae* was generated by homologous recombination (40). Heterodiploid strains containing single locus knockouts of FAL1, DBP2 or DBP5 were purchased from Dharmacon (GE Life Sciences). Strains were transformed with URA3-marked plasmids containing the genetic locus of the DEAD-box protein of interest and selected for on Ura- synthetic media. Strains were then sporulated by carbon starvation (1% potassium acetate, 0.1% yeast extract and 0.05% dextrose) for 5 days, released from their asci by lyticase treatment (Sigma-Aldrich) and dispersed by sonication (18). Haploid strains containing KanMX-cassette knockouts of FAL1, DBP2 or DBP5 at the genetic locus complemented with URA3-marked plasmids containing FAL1, DBP2 or DBP5 were selected on geneticin and Ura- synthetic complete media. Strains were confirmed to be unable to grow on 5-fluoroorotic acid (5-FOA) synthetic complete media, then verified by plasmid isolation followed by sequencing. Mating type was determined by complementation with MATa (his2-) and MATalpha (his2-) tester strains (gift from David Morgan, UCSF) on Ura- His- synthetic complete media. ES mutants were generated by site-directed mutagenesis in a HIS3-marked plasmid, transformed into yeast and counter selected using 5-FOA and His- synthetic complete media. Strains were verified by plasmid isolation followed by sequencing and subsequently grown in YPD media. Growth experiments are 10-fold dilutions from cultures grown to OD ∼1.

### Mass spectrometry assay

DDX3 (132–406) wild type (WT) or S228C (250 nM unless otherwise noted), DDX3 (132–607) (500 nM), RIG-I (230–795) (500 nM) or v-Src (250 nM) in 10 mM Tris (pH 7.5), 100 mM NaCl and 1 mM MgCl_2_ was incubated with compounds at 4°C. The extent of modification at various time points was determined by whole protein mass spectrometry using a Waters Acquity UPLC/G2-XF QTOF. Reported data points are single-replicates from three independent experiments (Figure 3B, Supplementary Figures S2E, S3A and C) or means of three independent experiments with error bars representing the standard error (Supplementary Figure S2A–C). Curves are fit using Prism (GraphPad).

### Differential scanning fluorimetry

Differential scanning fluorimetry (DSF) was performed as described (41) using 4 μM DDX3 (132–406) in a reaction buffer containing 20 mM HEPES (pH 7.5), 150 mM NaCl and 5 mM MgCl_2_. Reaction mixtures were incubated for 5 min prior to the start of the assay. Results are the average of three independent experiments with error bars representing the standard error of the mean. Curves are fit using Prism (GraphPad).

### In gel fluorescence

Purified DDX3^ES^ (132–607) or HA-eIF4A^ES^ yeast lysates were incubated with adenosine-5′-monophosphate (AMP)-acrylate followed by N-propargylmaleimide (NPM). Reactions were quenched with 0.5 mM β-mercaptoethanol, then TAMRA-N_3_, Tris[(1-benzyl-*1H*-1,2,3-triazol-4-yl)methyl]amine, sodium dodecyl sulphate, TCEP and CuSO_4_ were added and the reaction was allowed to proceed for 1 h. Samples were separated by sodium dodecyl sulphate-polyacrylamide gel electrophoresis and in gel fluorescence was measured by Typhoon Imager. Total protein levels were assessed by Coomassie staining. For immunoprecipitated protein samples, immunoprecipitation was performed after addition of β-mercaptoethanol quench, and samples were analyzed by immunoblot to ensure equivalent loading.

### X-ray crystallography

Purified DDX3^ES^ (132–607) was incubated with 100 μM AMP-acrylamide at 4°C until completely labeled, as judged by liquid chromatography-mass spectrometry (LC-MS). Protein was then applied to a Superdex 200 gel filtration column equilibrated in 20 mM HEPES (pH 7.5), 500 mM NaCl, 10% (v/v) glycerol and 0.5 mM TCEP and fractions containing pure protein were flash frozen in liquid nitrogen for storage. Conditions for crystallography were previously described (42). Data were collected at Beamline 8.2.2 of the Advanced Light Source (LBNL, Berkeley, CA). Data were indexed and integrated using iMosflm (43), scaled using Scala (44), phased using molecular replacement with PHASER (45) using Protein Data Bank (PDB) 5E7J as a search model and refined and built using PHENIX (46) and Coot (47). Structures were visualized with PyMOL (48).

### Chemical synthesis

For complete synthetic information, see Supplementary Data.

## RESULTS

### Low conservation residue of the P-loop as a site for electrophile-sensitive mutation

The nucleotide-binding pocket of DEAD-box proteins and ATPases, in general, is highly conserved and recalcitrant to mutation (4). In order to identify a chemically targetable and functionally silent mutation in the ATP-binding pocket, a structural alignment of all human DEAD-box proteins was performed. We identified the third residue of the P-loop/Walker A-motif (Figure 1B, red arrow) to be of lower conservation than surrounding nearly invariant residues. This residue lies ∼5–6Å from the α- and β-phosphates of ATP and forms a hydrogen bond with a single phosphate oxygen (Figure 1B). Although no human DEAD-box proteins natively express a cysteine at this position, several members of the structurally related DExH-box protein family do and cysteine is the third most common amino acid at this position across all human RNA helicases (Supplementary Figure S1A–C). We hypothesized that DEAD-box proteins could tolerate mutation of this position to cysteine to create an ES helicase mutant.

### Electrophile-sensitive DEAD-box proteins are functional

**Figure 2.**
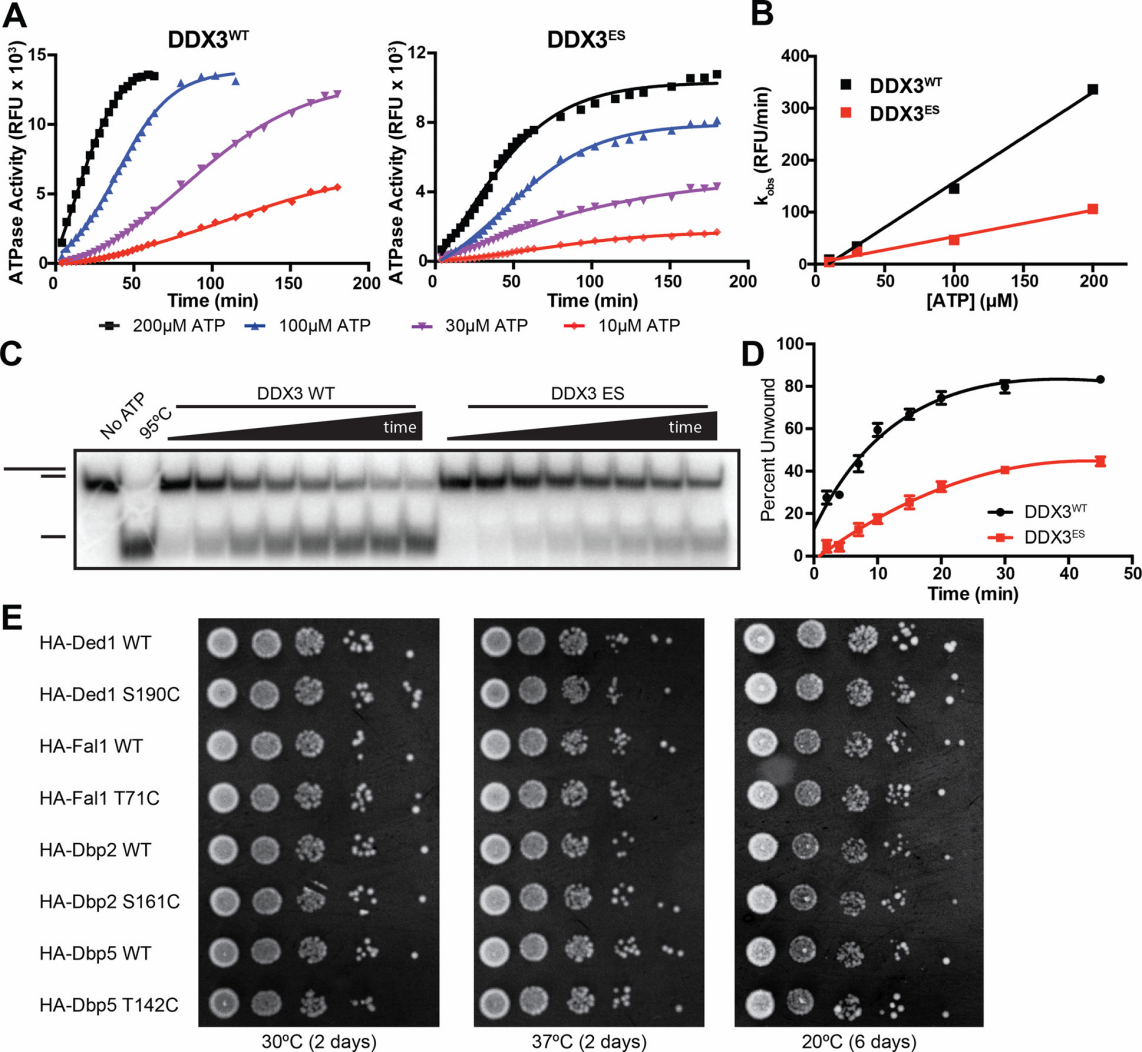
Electrophile-sensitive DEAD-box proteins retain biochemical and cellular function. (**A**) RNA-dependent ATPase activity of DDX3^WT^ and DDX3^ES^ (S228C) at four concentrations of ATP. (**B**) Comparison of the observed rate of RNA-dependent ATP hydrolysis by DDX3^WT^ and DDX3^ES^. (**C**) Representative RNA duplex unwinding assay comparing the activity of DDX3^WT^ and DDX3^ES^. (**D**) Quantification of the fraction of RNA duplex unwound by DDX3^WT^ and DDX3^ES^. (**E**) Serial dilutions of log-phase cultures of budding yeast strains expressing wild-type or ES DEAD-box proteins grown at permissive (30°C) or restrictive (20 and 37°C) temperatures.

To test if introduction of a cysteine residue in the ATP pocket (the putative ES-mutant) was tolerated in DEAD-box proteins, we expressed and purified wild-type and ES-versions of human DDX3 (residues 132–607) (42). Since the ES-mutation (S228C in DDX3) lies within the ATP-binding pocket, we first tested if DDX3^ES^ retained RNA-dependent ATPase activity. Indeed, both DDX3^WT^ and DDX3^ES^ hydrolyzed ATP in the presence of duplex RNA (Figure 2A), although DDX3^ES^ shows a 3-fold reduction in activity compared to the wild-type (Figure 2B).

DEAD-box proteins hydrolyze ATP to remodel RNA and RNA–protein complexes (6). To test if ES mutants retained this function, we utilized an RNA duplex unwinding assay that monitors the separation of a ^32^P-labeled 12-mer RNA oligonucleotide from a non-labeled 32-mer (37). ES DDX3 retains ATP-dependent RNA-unwinding activity, although the rate of unwinding is 3-fold lower than wild-type (Figure 2C and D). A similar reduction in RNA unwinding activity is observed in the ES version of closely related yeast Ded1 (Supplementary Figure S1D and E). In a third example, the ES mutant of yeast Dbp2 shows no reduction in RNA unwinding activity (Supplementary Figure S1F), suggesting a differential effect of this mutation across the DEAD-box family. These results indicate that three DEAD-box proteins expressing a non-natural cysteine residue in the nucleotide-binding pocket are biochemically functional.

To assess whether ES mutants of four different DEAD-box proteins (Ded1, Fal1, Dbp2 and Dbp5) are able to substitute for their WT counterpart *in vivo*, we determined their ability to rescue the loss of essential DEAD-box protein genes DED1, FAL1, DBP2 and DBP5 in *S. cerevisiae* (49–54). Yeast expressing ES versions of these DEAD-box proteins under endogenous promoters on extrachromosomal centromeric plasmids showed normal growth at permissive and restrictive temperatures (Figure 2E), in contrast to previous AS mutants (Supplementary Figure S1G). Taken together, these results demonstrate that ES DEAD-box proteins are functional biochemically and *in vivo*.

### Synthesis of electrophile-sensitive DEAD-box helicase inhibitors from AMP

To our knowledge there are currently no high-affinity ATP-competitive inhibitors of any RNA helicase. This limitation hindered our previous efforts to develop a chemical genetic method for the inhibition of the DEAD-box proteins using an AS strategy (27). Without a good drug-like starting point, we turned to nucleotide mimetics. Previous work identified AMP as the minimal component of ATP required for DEAD-box protein binding, since AMP is able to decrease RNA duplex unwinding by the DEAD-box protein Ded1 while adenosine is not (55). This work also found that AMP shows increased potency of inhibition of a subset of DEAD-box proteins as compared to ADP. These data illustrate the importance of the phosphate–P-loop electrostatic interactions for ligand binding. Therefore, we sought to develop novel high-affinity chemical probes by preserving these interactions while correctly positioning an electrophile for reaction with the engineered cysteine residue.

**Figure 3.**
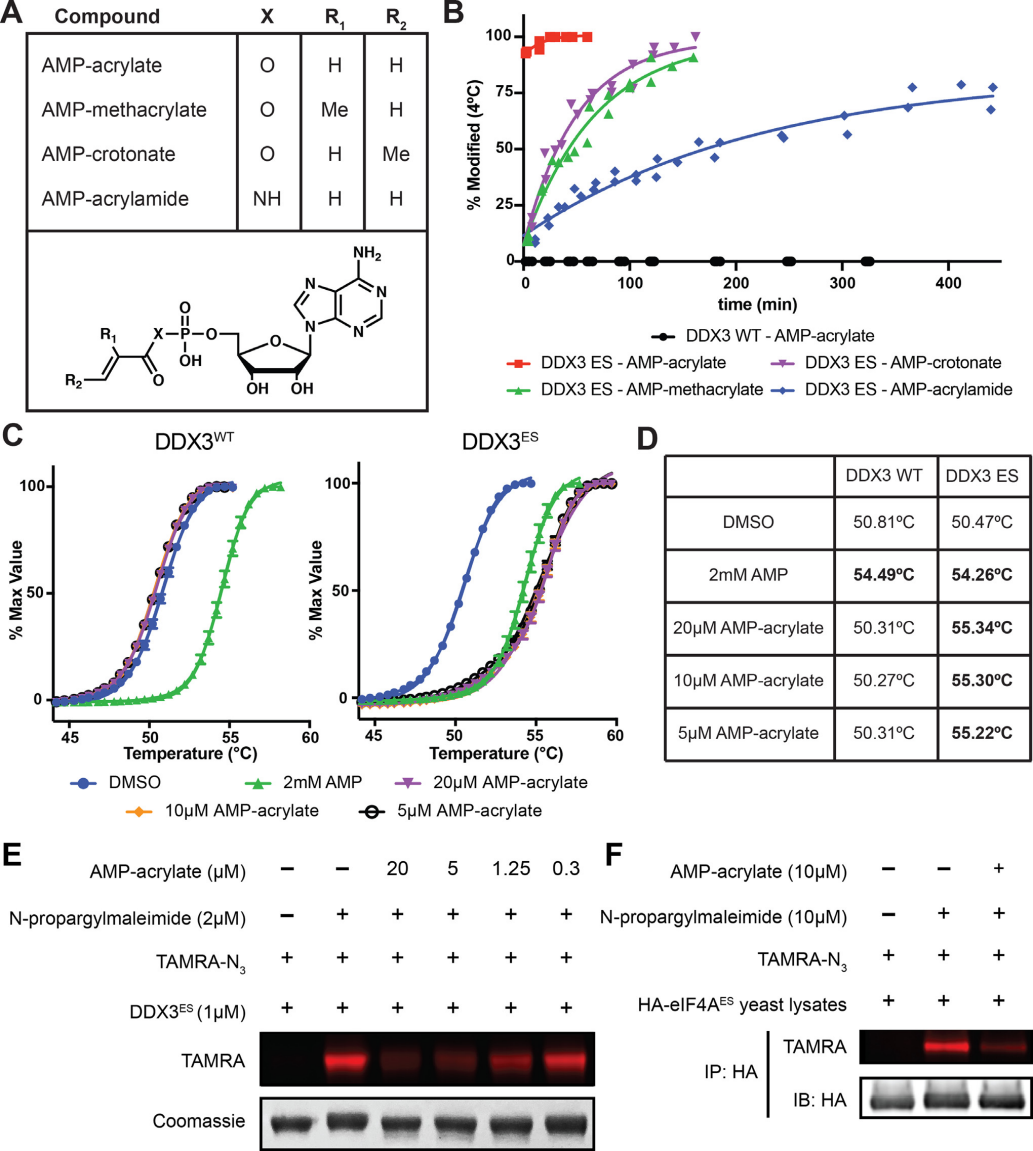
AMP-acrylate and derivatives potently and irreversibly bind ES DEAD-box proteins. (**A**) Structures of AMP-acrylate and derivatives used in this study. (**B**) Percent modification of DDX3 (132–406) WT and ES (S228C) incubated with 5 μM AMP-acrylate and derivatives at 4°C. (**C**) Differential scanning fluorimetry curves of DDX3 (132–406) WT and ES in the presence of AMP or 5, 10 or 20 μM AMP-acrylate. (**D**) Experimentally determined melting temperatures (*T*_m_) of DSF curves from (C) with significant differences from dimethyl sulfoxide (DMSO) in bold (*t*-test, *P* < 0.001). (**E**) Recombinant DDX3^ES^ (1 μM) treated with various concentrations of AMP-acrylate followed by 2 μM NPM. A click reaction is performed using TAMRA-N_3_ and the reaction is analyzed by in gel fluorescence (TAMRA). Loading is analyzed by Coomassie staining. (**F**) HA-eIF4A^ES^ lysates are treated with 10 μM AMP-acrylate followed by 10 μM NPM. HA-tagged proteins are isolated by immunoprecipitation. A click reaction is performed on immunoprecipitated proteins using TAMRA-N_3_, and the reaction is analyzed by in gel fluorescence (TAMRA). Loading is analyzed by immunoblot.

The simplest molecule with both of these characteristics is AMP-acrylate (Figure 3A), which appends a cysteine-reactive Michael acceptor from the phosphate of AMP through a phosphoester linkage. To test whether these electrophiles bind covalently to DDX3, we first constructed a truncated mutant (residues 132–406) with improved characteristics for mass spectrometry, which allows for direct monitoring of the formation of a covalent adduct. Treating this optimized form of DDX3^ES^ with AMP-acrylate showed complete adduct formation within 5 min at 5 μM, while no detectable binding to DDX3^WT^ was observed after 5 h of incubation (Figure 3B). This modification is robust and rapid down to equimolar concentrations of AMP-acrylate and protein (Supplementary Figure S2A). AMP-acrylate derivatives (AMP-methacrylate and AMP-crotonate) and AMP-acrylamide (Figure 3A) displayed reduced rates of reactivity against DDX3^ES^ according to their expected reduced cysteine reactivity (Figure 3B) (56). We also appended an electrophile to the acyclic AMP-analog 9-(2-phosphonomethoxyethyl)adenine (PMEA, adefovir) (57) to yield adefovir-acrylate (Supplementary Figure S2B). Adefovir-acrylate displayed only slightly reduced kinetics of labeling compared to AMP-acrylate (Supplementary Figure S2C), showing acyclic nucleotide analogs are also capable of labeling ES DEAD-box proteins. Thus, the high rate of reactivity of AMP-acrylates against ES DDX3 suggests that the nucleophile–electrophile pair are properly oriented within the ATP-binding site and the sparing of wild-type DDX3 shows that this reactivity is dependent on the presence of the engineered cysteine.

### AMP-acrylates undergo two-step reaction with electrophile-sensitive DEAD-box proteins

Although AMP-acrylates rapidly and irreversibly labeled DDX3^ES^ in 5 min at low micromolar doses, inspection of this reaction after longer periods of time surprisingly showed a loss of the full mass adduct (+ 401 Da) and the formation of a new adduct of + 54 Da (Supplementary Figure S2D and E). This adduct is not observed with AMP-acrylamide (data not shown). Carboxylic acids can be activated for nucleophilic attack in biological systems through formation of an acyl-phosphate, and acyl-phosphates are chemoselective reagents for acylation of basic amines (58), including the catalytic lysine of kinases (59,60). We hypothesized that the + 54 Da adduct is the result of addition of an active site nucleophile (for example K230 in DDX3) into the acyl-phosphate bond of AMP-acrylate and subsequent elimination of AMP (Supplementary Figure S2F). The phosphoramidate P–N bond is not labile under physiological conditions, consistent with the finding that the second step does not occur with AMP-acrylamide. Indeed, mutation of K230 in DDX3^ES^ reduced the rate of formation of this +54 Da state (Supplementary Figure S2E). That this mutation did not fully abrogate the formation of this + 54 Da adduct suggests that other nucleophilic residues in the nucleotide-binding site may additionally contribute to this reaction.

### AMP-acrylate maintains normal nucleotide–protein interactions with electrophile-sensitive DEAD-box proteins

Nucleotide binding to the DEAD-box protein active site requires numerous interactions that stabilize a specific enzyme conformation. To test if AMP-acrylates maintain these interactions upon covalent modification of ES enzymes, we used DSF to assess protein stabilization after compound binding (41). Saturating concentrations of AMP stabilizes DDX3^WT^ and DDX3^ES^ to the same extent (Figure 3C and D), again showing the ES mutation does not significantly disrupt nucleotide binding. However, only DDX3^ES^ is stabilized by AMP-acrylate (Figure 3C and D). This confirms we have developed a chemical probe with specific reactivity to ES DEAD-box proteins. That the magnitude of stabilization by AMP-acrylate is as large as the stabilization by saturating concentrations of AMP suggests that compound binding has preserved native protein–ligand interactions.

### AMP-acrylates modify electrophile-sensitive DEAD-box proteins in lysates

To evaluate the specificity of AMP-acrylates for ES DEAD-box proteins versus other ATP-binding proteins, we tested the rate of modification of the ATPase RIG-I and the kinase v-Src by AMP-acrylate (Supplementary Figure S3A). Despite natively expressing a P-loop cysteine, RIG-I shows slow modification by AMP-acrylate. Similarly, v-Src, which was previously shown to react with nucleotide-acrylates through alkylation of the catalytic lysine (60), shows <50% modification after 1 h (Supplementary Figure S3A). These results demonstrate that AMP-acrylates do not indiscriminately modify ATP-binding proteins.

Next, we assessed the reactivity of AMP-acrylates against cysteines in the proteome using N-propargylmaleimide (NPM) (Supplementary Figure S3B) as a semi-selective cysteine probe. At low doses, NPM specifically reacts with DDX3^ES^ over DDX3^WT^ (Supplementary Figure S3C), likely because the ES mutation mimics the N-ethylmaleimide-reactive cysteine of NSF (33). Therefore, NPM can be used as a semi-selective occupancy probe of ES DEAD-box proteins. Recombinant DDX3^ES^ exhibits a dose responsive decreased in NPM labeling after treatment with AMP-acrylate (Figure 3E), showing these chemical probes react with the same cysteine in DDX3^ES^. However, while yeast lysates treated with AMP-acrylate do not show a global decrease in NPM binding (Supplementary Figure S3D), labeling of HA-eIF4A^ES^ by NPM is competed by AMP-acrylate treatment (Figure 3F). These results indicate that AMP-acrylates target ES DEAD-box proteins in lysates without broadly reacting with endogenous cysteines in the proteome.

### A 3.0 Å crystal structure of DDX3^ES^ bound to AMP-acrylamide

**Figure 4.**
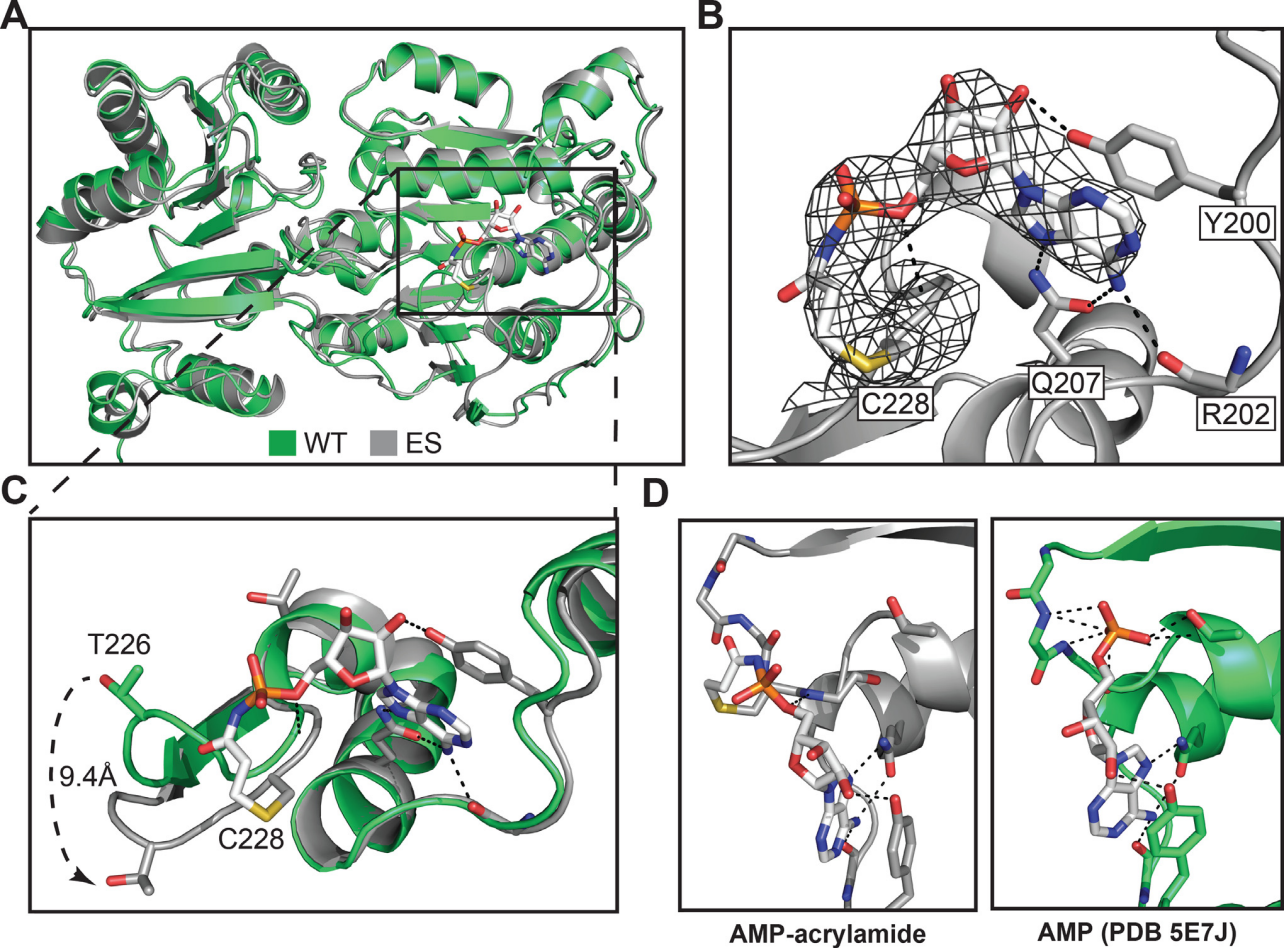
Crystal structure of AMP-acrylamide bound to ES DDX3. (**A**) Overall structure of AMP-acrylamide bound to DDX3^ES^ (gray) aligned to AMP-bound DDX3 (green, PDB ID: 5E7J). (**B**) AMP-acrylamide bound to the nucleotide-binding site of DDX3 with conserved hydrogen-bonding interactions (black lines) with Y200, R202 and Q207. AMP-acrylamide 2mF_o_-DF_c_ difference map is shown (black mesh, 1.5σ). (**C**) Alignment of AMP-acrylamide- (gray) and AMP-bound (green) structures of DDX3 shows flattening of the P-loop in AMP-acrylamide-bound structure including an 9.4 Å shift in T226. (**D**) Comparison of the coordination of the phosphoramidate of AMP-acrylamide (gray) to the coordination of the phosphate of AMP (green) by DDX3 (PDB 5E7J) (dashed lines are predicted hydrogen bonds).

To better understand the binding of AMP-acrylates to ES DEAD-box proteins, we solved the crystal structure of DDX3 (132–607) S228C bound to AMP-acrylamide to 3.0 Å (Supplementary Table S1). The overall protein structure is highly similar to the previously published structure of DDX3 bound to AMP (42), with a root mean squared deviation of 1.265 Å (Figure 4A), as expected from its similar thermal stability (Figure 3C and D). AMP-acrylamide can be fit unambiguously to its density and clearly binds cysteine-228 through a covalent bond (Figure 4B). The adenine of AMP-acrylamide maintains several hydrogen-bonding interactions with the Q-motif of DDX3 including interactions with glutamine-207 and the backbone of arginine-202, as well as pi-stacking with tyrosine-200 (Figure 4B). The covalent linkage significantly re-orders the P-loop into a conformation that to our knowledge has not previously been observed in nucleotide-bound structures of DEAD-box proteins. This results in a 9.4 Å shift in the side chain of T226 as compared to AMP-bound DDX3 (Figure 4C). In addition, the phosphoramidate of AMP-acrylamide is also displaced more than 3 Å out of the phosphate-binding pocket as compared to the phosphate of AMP so that only the 5′-oxygen hydrogen bonds with the P-loop backbone, whereas the phosphate of AMP typically makes numerous electrostatic interactions (Figure 4D).

### AMP-acrylate specifically inhibits duplex unwinding by electrophile-sensitive DEAD-box proteins

In addition to specifically binding to ES DEAD-box proteins, we asked whether AMP-acrylate binding inhibits DEAD-box protein function. Previously, compounds were identified that reduced DDX3 ATPase activity in a mutant selective manner, but did not inhibit its duplex unwinding activity (27). This is likely due to the large stoichiometric excess of enzyme required for the single-turnover nature of the duplex unwinding assay while the ATPase assay utilized high substrate–enzyme ratios. Therefore, AMP-acrylates were tested for their ability to inhibit duplex unwinding by wild-type and ES DEAD-box proteins.

**Figure 5.**
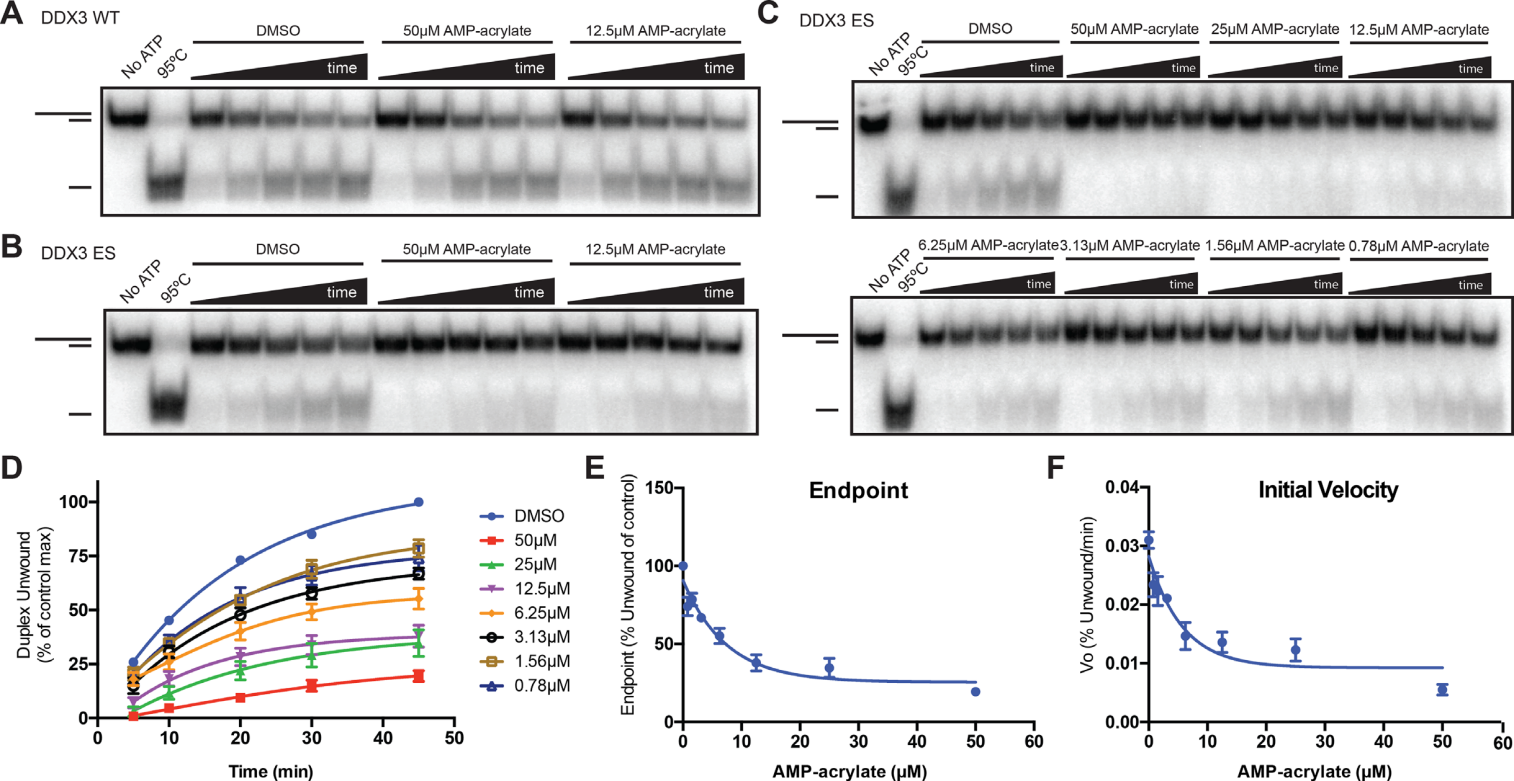
AMP-acrylates inhibit ES DEAD-box proteins. (**A** and **B**) RNA duplex unwinding by DDX3^WT^ (A) and DDX3^ES^ (S228C) (B) in the presence of 50 and 12.5 μM AMP-acrylate. (**C** and **D**) RNA duplex unwinding by DDX3^ES^ in the presence of serial dilutions of AMP-acrylate starting at 50 μM (C) with quantification of three independent experiments (D). (**E** and **F**) Effect of AMP-acrylate of the endpoint (E) and rate (F) of RNA duplex unwinding by DDX3^ES^.

While AMP-acrylate shows no appreciable inhibition of wild-type DDX3 (Figure 5A), the duplex unwinding activity of DDX3^ES^ is significantly reduced (Figure 5B). This inhibition is dose-dependent (Figure 5C and D) down to equimolar ratios of probe–enzyme, consistent with our previous findings that equimolar amounts of AMP-acrylate stoichiometrically bind to DDX3^ES^ (Supplementary Figure S2A). Surprisingly, AMP-acrylate reduces both the endpoint (Figure 5E) and the rate (initial velocity, Figure 5F) of duplex unwinding by DDX3^ES^, in contrast to AMP, which largely showed endpoint depression of duplex unwinding by DDX3^WT^ (27). To confirm that these results are not confined to one ES mutant DEAD-box protein, we also show that duplex unwinding by ES Dbp2 (S161C) is reduced by AMP-acrylate, while Dbp2^WT^ is unaffected (Supplementary Figure S4A and B). These results demonstrate that AMP-acrylates are a novel chemical tool for the inhibition of biochemical activity of ES DEAD-box proteins.

## DISCUSSION

We have developed a strategy for the chemical genetic inhibition of DEAD-box proteins through covalent complementarity. After identifying a residue of low conservation in the P-loop of all DEAD-box proteins (Figure 1), we generated ‘electrophile-sensitive’ DEAD-box proteins with near wild-type biochemical activity and the ability to complement loss of essential alleles in yeast (Figure 2). We then developed a series of AMP-acrylates that rapidly and specifically bind and inhibit these ES DEAD-box proteins (Figures 3 and 5).

To our knowledge, the structure of AMP-acrylamide bound to DDX3^ES^ is the first crystal structure of a DEAD-box protein in complex with a small molecule inhibitor. The significant flattening of the P-loop observed in this structure is likely due to the torsion placed on this flexible loop by the tethered small molecule as it moves to maintain hydrogen bonds within the Q-motif. Interestingly, this distortion of the P-loop breaks the electrostatic interactions between the phosphate and the P-loop backbone, which are typically thought to drive nucleotide affinity. Since covalent compounds are hypothesized to undergo rapid reversible binding prior to covalent bond formation, we hypothesize that the initial binding event of AMP-acrylates by ES DEAD-box proteins is driven by normal electrostatic interactions between the phosphate and P-loop and that covalent bond formation disrupts the positioning of the protein and small molecule to the final position observed in the crystal structure. This phenomenon was also observed with covalent inhibitors of Src kinase, which lost hinge hydrogen bonding contacts upon covalent bond formation (61). We cannot, however, rule out the possibility that this is an artifact of the rigidity of the amide bond of AMP-acrylamide and not representative of AMP-acrylates as a whole. This could explain the reduced rate of covalent modification observed by AMP-acrylamide as compared to AMP-acrylates (Figure 3B). Nonetheless, we expect the reordering of the P-loop observed upon binding of this compound to strongly antagonize the closed (active) conformation of DDX3.

Since we found that AMP-acrylates modify ES DEAD-box proteins in lysates (Figure 3F), we anticipate significant potential utility for these probes in cell-free systems in which DEAD-box proteins are often studied, including assays of spliceosomal assembly and function (62–64), ribosome biogenesis (65,66) and translation (67–70). The current AMP-acrylate inhibitors are likely unable to cross the plasma membrane and thus cannot be used in whole-cell systems. These problems can be addressed through various pro-drug strategies developed for monophosphate- and phosphonate-containing nucleotide-reverse transcriptase inhibitors (57,71,72) or through development of more ‘drug-like’ phosphate-mimics. Acyclic derivatives of AMP-acrylate, like adefovir-acrylate (Supplementary Figure S2B and C), could additionally provide a path for improving the pharmacological properties of ES DEAD-box protein inhibitors. Continued screening for small molecules that bind the DEAD-box protein nucleotide-binding site may yield novel chemotypes with improved pharmacological properties that can be modified into ES inhibitors. AMP-acrylates could be used as occupancy probes to aid in the identification of nucleotide-competitive compounds (73).

AMP-acrylates developed in this work are notable because of their extremely fast binding to ES DEAD-box proteins and their selectivity for ES over wild-type enzymes (Figure 3B). The rapid kinetics of binding and inhibition allows for little or no pre-treatment of compounds, thus mitigating off-target effects. These effects can be additionally ruled out through the use of wild-type controls, which appear to be completely spared from inhibition at doses of AMP-acrylate up to 50 μM (Figure 5A). Although previous studies have identified AMP as an inhibitor of DEAD-box proteins at doses as low as 10 μM (27), we hypothesize that AMP-acrylates have reduced reversible affinity for the nucleotide-binding site of DEAD-box proteins because of the added electrophilic moiety and reduced formal charge. That no reactivity was observed between AMP-acrylates and wild-type enzymes additionally suggest that the cysteine reaction is required for addition into the phosphoanhydride bond (Supplementary Figure S2D and F). This is likely due to the fast kinetics of the cysteine reaction and slow kinetics of addition into the phosphoanhydride, which is only accessible upon covalent tethering of the compound into the active site. Therefore, AMP-acrylates show improved affinity for ES DEAD-box proteins due to covalent complementarity and reduced binding to wild-type enzymes. These characteristics make these probes ideal for the study of complex biological processes with multiple ATPases in which DEAD-box proteins often function.

While no DEAD-box proteins natively express a P-loop cysteine, RIG-I, a closely related protein of the RLR family of RNA helicases (74), and several DExH-box proteins express endogenous cysteines at this position. This raises the intriguing possibility that small molecules related to the chemical tools developed in this work could function as native chemical inhibitors of these enzymes. Additionally, since DExH-box proteins exhibit unselective nucleotide base binding and do not contain a Q-motif (75), changing the nucleotide base of AMP-acrylates may yield compounds with heightened specificity for natively electrophile-sensitive DExH-box proteins. The different orientation of the nucleotide base relative to the triphosphates in DExH-box proteins may also result in altered binding by acyclic AMP derivatives such as adefovir-acrylate.

Although our work establishes a chemical genetic strategy in DEAD-box proteins only, the high conservation of the P-loop and nucleotide-binding site suggests that this strategy may work in other RNA helicases and ATPases in general. In fact, all other families of ATPases (AAA+, ABC transporters, etc.) contain family members with native cysteines at this position (Supplementary Figure S1A). This implies that the overall ATPase fold can accept the ES mutation and suggests that the strategy developed in this work may have implications outside the DEAD-box protein family. The presence of related enzymes with native cysteines at this position also suggests that the AMP-acrylate may react with proteins outside of the RNA-helicase family. Our results using NPM rule out indiscriminant cysteine reactivity across the proteome, yet other modifications to AMP-acrylate such as the acyclic adefovir-acrylate may obviate any remaining ‘off-target’ activities. Although AMP-acrylates likely have utility beyond DEAD-box proteins, we expect that novel probes will be required for the specific inhibition of the diverse ATPase families. This will allow for the specific chemical inhibition of nearly 500 human enzymes, encompassing nearly every biological process.

## DATA AVAILABILITY

Atomic coordinates and structure factors for the reported crystal structure have been deposited with the Protein Data Bank under accession number 6CZ5.

## Supplementary Material

Supplementary Data
