# Supplementary material for: Chemical genetic inhibition of DEAD-box proteins using covalent complementarity
Source: Nucleic Acids Res. 2018 Aug 8;46(17):8689–99. doi: 10.1093/nar/gky706 (PMC6158709; doi:10.1093/nar/gky706)
Supplement: Supplementary Data [file gky706_supplemental_files.pdf]

Supplementary Figure 1

A

Enzyme Superfamily	Family Members with Cysteine at this Position
RNA Helicases	RIG-I, DHX9/RHA, DHX30, DHX32, DHX34, DHX35, DHX36, DHX57
DNA Helicases	HELB
AAA+ ATPases	p97/VCP, APAF1, VWA8, RAD51B, RAD17, NVL, ATP6V1A, PSMC4, NSF, PEX6, ATAD1, RNF213, SPATA5, MDN1, SPG7, BCS1L, GNN, WRNIP1
ABC Transporters	MDR1, MDR3, MRP, MRP7, CMOAT2, ABCB5, ABCB11, ABCC8, ABCC9, ABCD1, ABCD2, ABCD3, ABCG8
Kinesins	KIF19
Dyneins	DYNC2H1
Total proteome	43/418 total ATPases

B

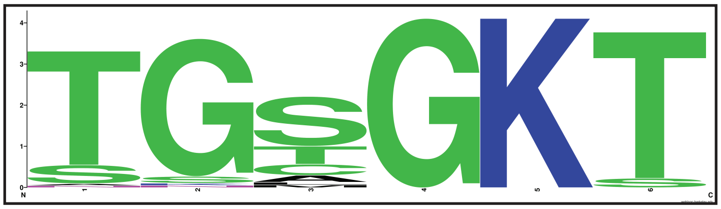

C

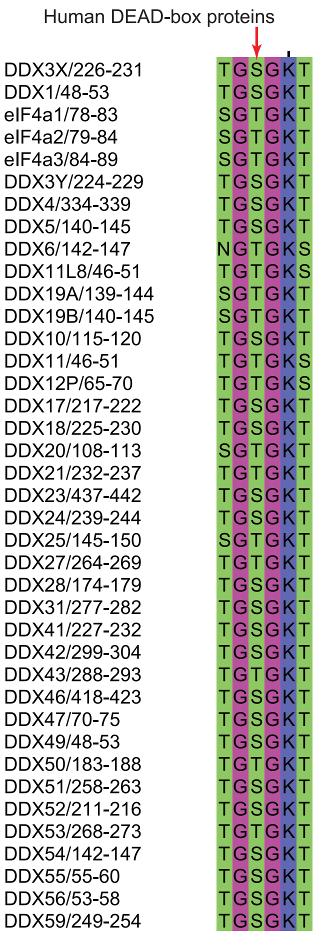

D

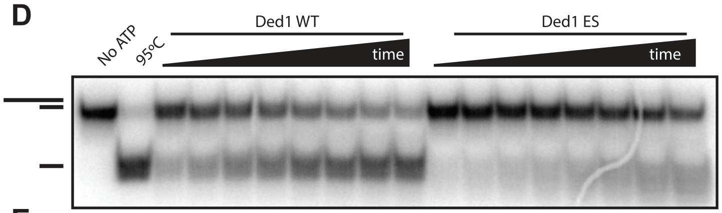

E

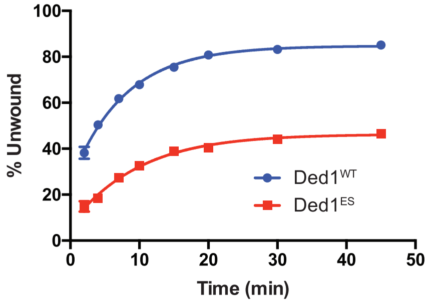

F

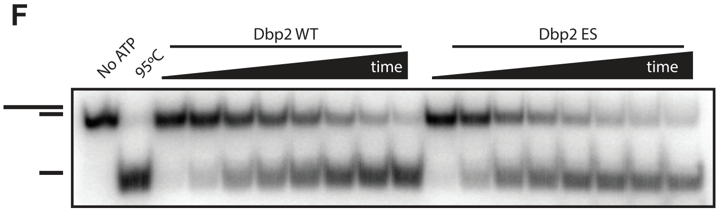

G

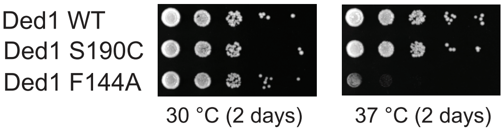

Supplementary Figure 2

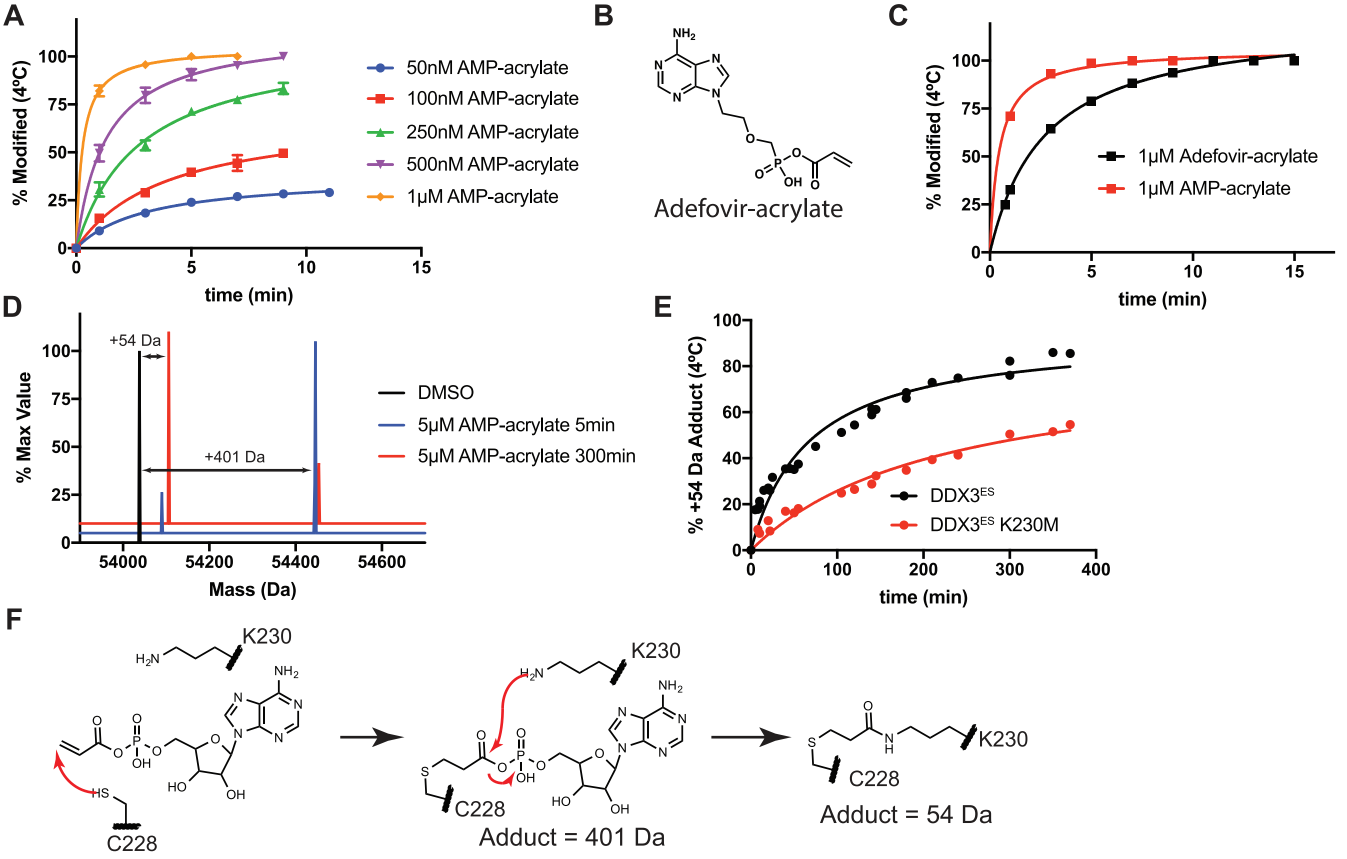

Supplementary Figure 3

A

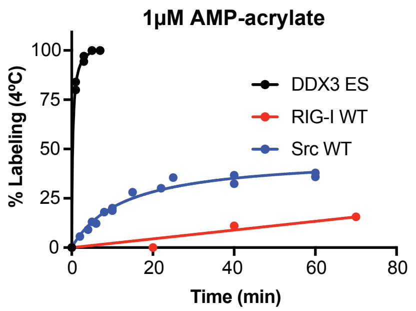

B

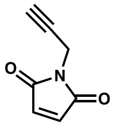

N-propargylmaleimide
(NPM)

C

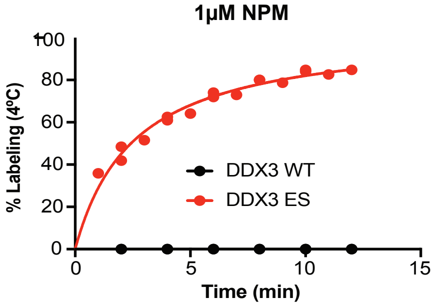

D

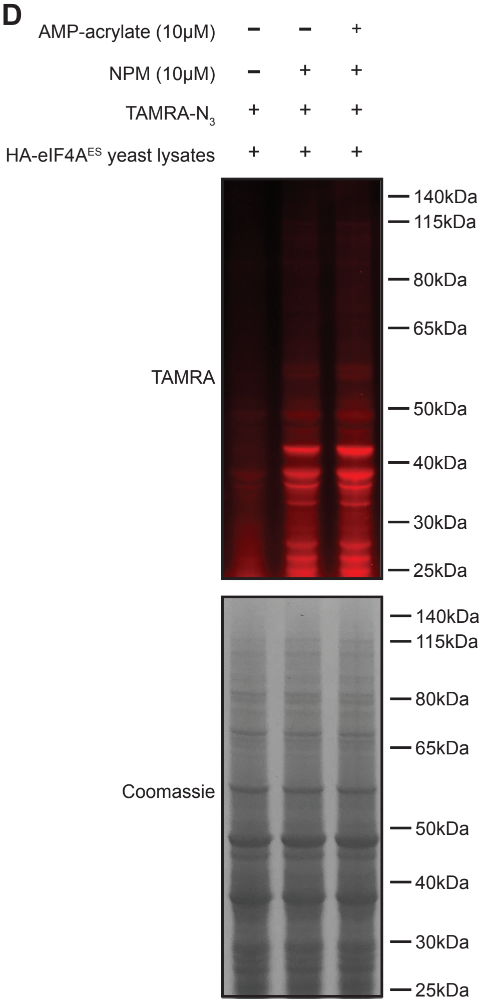

Supplementary Figure 4

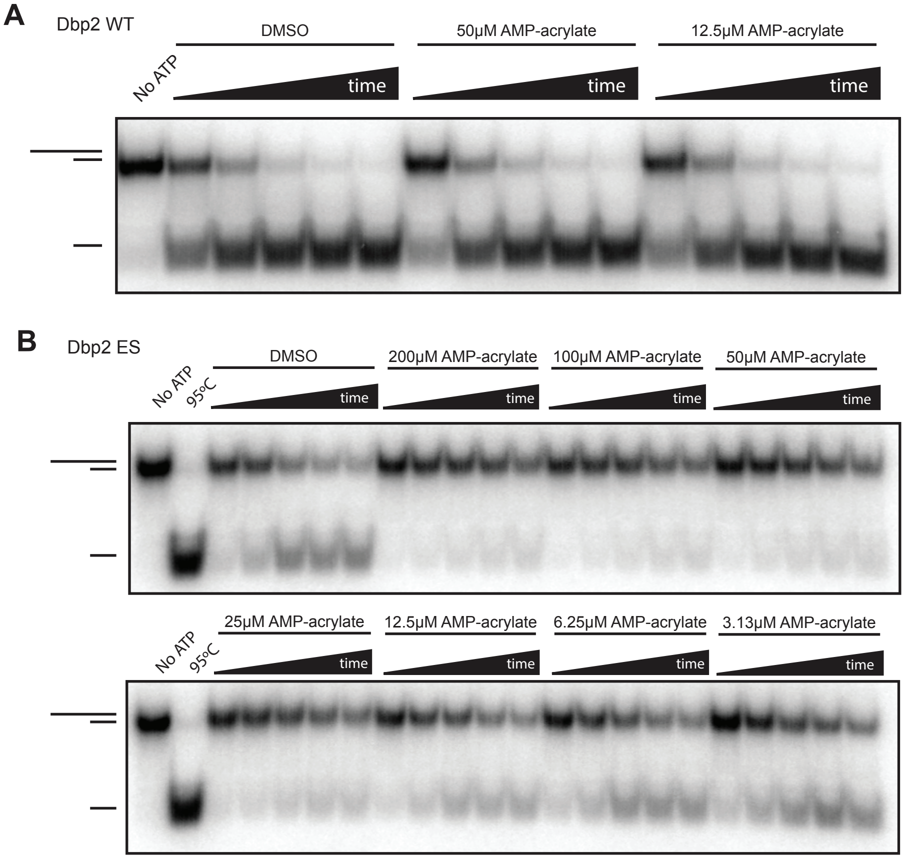

SUPPLEMENTARY INFORMATION

Supplementary Table 1. Data collection and refinement statistics.

	DDX3 (132-607) – AMP-acrylamide
**Data collection**	
Space group	P 21 21 21
Cell dimensions	
a, *b*, *c* (Å)	53.96, 101.09, 105.69
α, *β*, *γ* (°)	90, 90, 90
Resolution (Å)	73.05-3.00 (3.16-3.00)[a]
R_{merge}, R_{meas}, and R_{pim}	0.121 (1.089), 0.135 (1.201), 0.057 (0.500)
$I/\sigma(I)$	8.5 (1.6)
$CC_{1/2}$	0.996 (0.766)
Completeness (%)	100.0 (100.0)
Redundancy	5.4 (5.6)
**Refinement**	
Resolution (Å)	73.05-3.00
No. reflections	12100
R_{work} / R_{free}	0.2219/0.2667
No. atoms	
Protein	3378
Ligand/ion (specify/describe)	27
Water	0
B factors	
Protein	87.39
Ligand/ion	107.20
R.m.s. deviations	
Bond lengths (Å)	0.002
Bond angles (°)	0.50

[a] Values in parentheses are for highest-resolution shell.

Supplementary Table 2: Yeast strains used in this study

**Strain**	**Genotype**	**Source**
Ded1 WT	*MAT**a** his3Δ1 leu2Δ0 met15Δ0 ura3Δ0* ded1::KanMX pKJB001	Hilliker A et al. (2011) (1)
HA-Ded1 WT	*MAT**a** his3Δ1 leu2Δ0 met15Δ0 ura3Δ0* ded1::KanMX pKJB002	This study
HA-Ded1 S190C	*MAT**a** his3Δ1 leu2Δ0 met15Δ0 ura3Δ0* ded1::KanMX pKJB003	This study
Fal1 HetDip	*MAT**a**/MAT**alpha** his3Δ1/his3Δ1 leu2Δ0/leu2Δ0* MET15*/met15Δ0* LYS2*/lys2Δ*0 *ura3Δ0/ura3Δ0* FAL1/fal1::KanMX	GE Dharmacon (Clone ID: 23960)
Fal1 WT	*MAT**alpha** his3Δ1 leu2Δ0 ura3Δ0* fal1::KanMX pKJB004	This study
HA-Fal1 WT	*MAT**alpha** his3Δ1 leu2Δ0 ura3Δ0* fal1::KanMX pKJB005	This study
HA-Fal1 T71C	*MAT**alpha** his3Δ1 leu2Δ0 ura3Δ0* fal1::KanMX pKJB006	This study
Dbp2 HetDip	*MAT**a**/MAT**alpha** his3Δ1/his3Δ1 leu2Δ0/leu2Δ0* MET15*/met15Δ0* LYS2*/lys2Δ*0 *ura3Δ0/ura3Δ0* DBP2/dbp2::KanMX	GE Dharmacon (Clone ID: 27822)
Dbp2 WT	*MAT**a** his3Δ1 leu2Δ0 ura3Δ0* dbp2::KanMX pKJB007	This study
HA-Dbp2 WT	*MAT**a** his3Δ1 leu2Δ0 ura3Δ0* dbp2::KanMX pKJB008	This study
HA-Dbp2 S161C	*MAT**a** his3Δ1 leu2Δ0 ura3Δ0* dbp2::KanMX pKJB009	This study
Dbp5 HetDip	*MAT**a**/MAT**alpha** his3Δ1/his3Δ1 leu2Δ0/leu2Δ0* MET15*/met15Δ0* LYS2*/lys2Δ*0 *ura3Δ0/ura3Δ0* DBP5/dbp5::KanMX	GE Dharmacon (Clone ID: 21822)
Dbp5 WT	*MAT**alpha** his3Δ1 leu2Δ0 ura3Δ0* dbp5::KanMX pKJB010	This study
HA-Dbp5 WT	*MAT**alpha** his3Δ1 leu2Δ0 ura3Δ0* dbp5::KanMX pKJB011	This study
HA-Dbp5 T142C	*MAT**alpha** his3Δ1 leu2Δ0 ura3Δ0* dbp5::KanMX pKJB011	This study
HA-eIF4A^{ES}	*MATa his3Δ1 leu2Δ0 met15Δ0 ura3Δ0* tif1::HA-Tif1 T70C tif2::URA3	This study

Supplementary Table 3: Plasmids used in this study

Plasmid	Description	Vector	Insert	Source
pKJB001	Ded1 WT	pRS416 (*URA3/CEN*)	DED1 (-106 to +2727)	Hilliker A et al. (2011)
pKJB002	HA-Ded1 WT	pRS413 (*HIS3/CEN*)	DED1 (-106 to +2727)/N-terminal HA-tag	This study
pKJB003	HA-Ded1 S190C	pRS413 (*HIS3/CEN*)	DED1 S190C (-106 to +2727)/N-terminal HA-tag	This study
pKJB004	Fal1 WT	pRS416 (*URA3/CEN*)	FAL1 (-607 to +1826)	This study
pKJB005	HA-Fal1 WT	pRS413 (*HIS3/CEN*)	FAL1 (-607 to +1826)/N-terminal HA-tag	This study
pKJB006	HA-Fal1 T71C	pRS413 (*HIS3/CEN*)	FAL1 T71C (-607 to +1826)/N-terminal HA-tag	This study
pKJB007	Dbp2 WT	pRS416 (*URA3/CEN*)	DBP2 (-757 to +4107; Δintron)	This study
pKJB008	HA-Dbp2 WT	pRS413 (*HIS3/CEN*)	DBP2 (-757 to +4107; Δintron)/N-terminal HA-tag	This study
pKJB009	HA-Dbp2 S161C	pRS413 (*HIS3/CEN*)	DBP2 S161C (-757 to +4107; Δintron)/N-terminal HA-tag	This study
pKJB010	Dbp5 WT	pRS416 (*URA3/CEN*)	DBP5 (-1029 to +2462)	This study
pKJB011	HA-Dbp5 WT	pRS413 (*HIS3/CEN*)	DBP5 (-1029 to +2462)/N-terminal HA-tag	This study
pKJB012	HA-Dbp5 T142C	pRS413 (*HIS3/CEN*)	DBP5 T142C (-1029 to +2462)/N-terminal HA-tag	This study

SUPPLEMENTARY FIGURE LEGENDS

Supplementary Figure 1. Development of an 'electrophile-sensitive' mutation in DEAD-box proteins. A. Identification of human ATPases from diverse enzyme families that natively express a P-loop cysteine. **B.** Structural alignment of the P-loop of all human RNA helicases. **C.** Alignment of the P-loop of all human DEAD-box proteins to identify position of electrophile-sensitive mutation (red arrow). **D.** Representative RNA duplex unwinding assay comparing the activity of $Ded1^{WT}$ and $Ded1^{ES}$ (S190C). **E.** Quantification of the fraction of RNA duplex unwound by $Ded1^{WT}$ and $Ded1^{ES}$. **F.** Representative RNA duplex unwinding assay comparing the activity of $Dbp2^{WT}$ and $Dbp2^{ES}$ (S161C). **G.** Serial dilutions of log-phase cultures of budding yeast strains expressing wild-type, electrophile-sensitive (S190C), or analog-sensitive (F144A) Ded1 grown at permissive (30ºC) or restrictive (37ºC) temperatures.

Supplementary Figure 2. AMP-acrylates undergo two-step reaction with electrophile-sensitive DEAD-box proteins. A. Percent modification of 125nM $DDX3^{ES}$ (S228C) by various concentrations of AMP-acrylate at 4ºC. **B.** Structure of Adefovir-acrylate. **C.** Comparison of the rate of modification of 250nM $DDX3^{ES}$ by 1µM AMP-acrylate and 1µM Adefovir-acrylate at 4ºC. **D.** Whole-protein mass spectrometry of $DDX3^{ES}$ with DMSO or 5µM AMP-acrylate at 4ºC showing formation of +401 Da adduct after 5min and +54 Da adduct after 300min. **E.** Quantification of the percentage of $DDX3^{ES}$ and $DDX3^{ES}$ K230M with +54 Da adduct over time. **F.** Proposed mechanism for the two-step reaction of $DDX3^{ES}$ with AMP-acrylates. The electrophile-sensitive mutation (C228 in DDX3) undergoes a Michael-addition into the electrophilic beta-carbon of the acrylate of AMP-acrylate (first-step). AMP is then eliminated from this adduct by addition of a nucleophilic amino acid such as lysine (K230 in DDX3; second-step) or arginine to form the final product.

Supplementary Figure 3. AMP-acrylates specifically modify electrophile-sensitive DEAD-box proteins in cell lysates. A. Comparison of the rate of modification of $DDX3^{ES}$, RIG-I WT, and Src WT by 1µM AMP-acrylate at 4ºC. **B.** Chemical structure of N-propargylmaleimide (NPM). **C.** Comparison of the rate of modification of $DDX3^{WT}$ and $DDX3^{ES}$ by 1µM NPM. **D.** HA-$eIF4A^{ES}$ lysates are treated with 10µM AMP-acrylate followed by 10µM NPM. A click reaction is performed using TAMRA-N_3 and the reaction is analyzed by in gel fluorescence (TAMRA). Loading is analyzed by coomassie staining.

Supplementary Figure 4. AMP-acrylates inhibit electrophile-sensitive DEAD-box proteins. A. RNA duplex unwinding by Dbp2 WT in the presence of 50µM and 12.5µM AMP-acrylate. **B.** RNA duplex unwinding by electrophile-sensitive Dbp2 (S161C) in the presence of serial dilutions of AMP-acrylate starting at 200µM.

CHEMICAL SYNTHESIS

Materials obtained commercially were reagent grade and were used without further purification. N-propargylmaleimide was purchased from Enamine BB. Reactions were monitored by thin layer chromatography (TLC) and/or mass spectrometry (LC-MS)

using a Waters Acquity UPLC/ESI-G2 XS QTOF. All NMR spectra were obtained on a Brucker 400 spectrometer.

Synthesis of intermediate 1

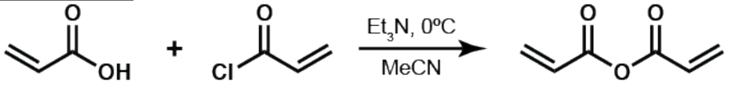

1

Acrylic anhydride (intermediate 1). Acrylic acid (Sigma-Aldrich, 205.7µL, 3.0mmol, 1.0eq) was added to a 20mL scintillation vial under Ar(g), followed by tetrahydrofuran (5mL) and triethylamine (418.1µL, 3.0mmol, 1.0eq). The reaction mixture was chilled to 0ºC and acryloyl chloride (242.4µL, 3.0mmol, 1.0eq) in tetrahydrofuran (1mL) was added dropwise. The reaction was allowed to warm to room temperature and stirred overnight. The reaction was filtered, evaporated to dryness, then dissolved in ethyl acetate (30mL) and extracted with a solution of saturated sodium bicarbonate. The organic fraction was dried with sodium sulfate, filtered, evaporated to dryness and the resulting oil was used without further purification.

General procedure for the synthesis of AMP-acrylate, AMP-crotonate, and AMP-methacrylate

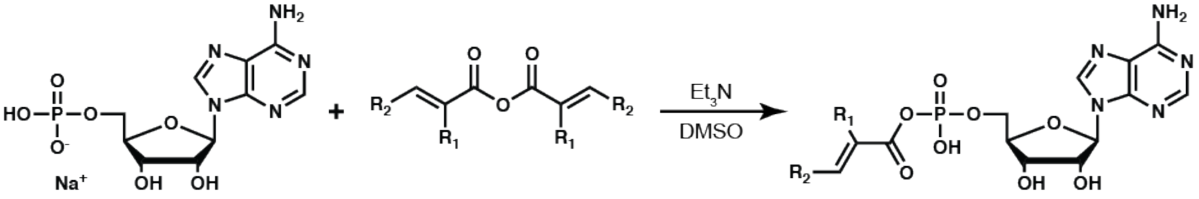

AMP-acrylates

In a 4mL scintillation vial, adenosine 5'-monophosphate sodium salt (Sigma, 25mg, 0.0720mmol) was dissolved in DMSO (1mL) and triethylamine (100µL,0.720mmol, 10eq) with sonication and stirring. In a separate vial, the appropriate symmetric acrylic anhydride (0.0792mmol, 1.1eq) was dissolved in DMSO (100µL) and added slowly to the reaction vial. After 30min, the reaction was quenched by addition of 1% formic acid (1mL) and purified by reverse phase HPLC (0-30% acetonitrile/water) to yield the pure compound.

acrylic (((2*R*,3*S*,4*R*,5*R*)-5-(6-amino-9*H*-purin-9-yl)-3,4-dihydroxytetrahydrofuran-2-yl)methyl phosphoric) anhydride (AMP-acrylate). White solid, 20.6mg, 71.2% yield. ^{1}H NMR (400MHz, D_2O) δ 8.48 (s, 1H), 8.33 (s, 1H), 6.31 (dd, *J* = 14.6, 3.5 Hz, 1H), 6.08 (d, *J* = 5.2 Hz, 1H), 6.01 – 5.89 (m, 2H), 4.74 (t, *J*= 5.2 Hz, 1H), 4.45 (t, *J* = 4.5 Hz, 1H), 4.31 (s, 1H), 4.20 (tq, *J* = 9.8, 6.4, 4.5 Hz, 2H). ^{31}P NMR (162MHz, D_2O) δ -7.32. ^{13}C NMR (100MHz, D_2O) δ 163.57 (d, *J* = 8.2 Hz), 150.49, 148.44, 145.49, 142.17, 134.56, 127.25 (d, *J* = 7.5 Hz), 118.58, 88.00, 83.91 (d, *J* = 8.7 Hz), 74.23, 70.22, 65.59 (d, *J* = 5.7 Hz). $[M+H]^+$ *m/z* calculated 402.0809 (100%), 403.0843 (14%), observed 402.0800 (100%), 403.0861 (12%).

(((2*R*,3*S*,4*R*,5*R*)-5-(6-amino-9*H*-purin-9-yl)-3,4-dihydroxytetrahydrofuran-2-yl)methyl phosphoric) (*E*)-but-2-enoic anhydride (AMP-crotonate). White solid,

13.66mg, 44.8% yield. ^{1}H NMR (400MHz, D_2O) δ 8.48 (s, 1H), 8.33 (s, 1H), 6.93 (m, 1H), 6.07 (d, J = 5.3 Hz, 1H), 5.63 (dt, J = 15.5, 1.9 Hz, 1H), 4.75 (t, J = 5.2 Hz, 1H), 4.45 (m, 1H), 4.31 (d, J = 2.9 Hz, 1H), 4.17 (pt, J = 5.6, 2.4 Hz, 2H), 1.74 (dd, J = 6.9, 1.6 Hz, 3H). ^{31}P NMR (162MHz, D_2O) δ -7.27. ^{13}C NMR (100MHz, D_2O) δ 163.80 (d, J = 8.2 Hz), 150.67, 150.36, 148.43, 145.36, 142.28, 121.00 (d, J = 7.4 Hz), 118.53, 88.01, 84.00 (d, J = 8.5 Hz), 74.17, 70.26, 65.53 (d, J = 5.8 Hz), 17.52. $[M+H]^+$ *m/z* calculated 416.0966 (100%), 417.0999 (15%), observed 416.0961 (100%), 417.0987 (12%).

(((2*R*,3*S*,4*R*,5*R*)-5-(6-amino-9*H*-purin-9-yl)-3,4-dihydroxytetrahydrofuran-2-yl)methyl phosphoric) methacrylic anhydride (AMP-methacrylate). White solid, 10.0mg, 32.9% yield. ^{1}H NMR (400MHz, D_2O) δ 8.46 (s, 1H), 8.32 (s, 1H), 6.07 (d, J = 5.3 Hz, 1H), 5.95 (s, 1H), 5.59 (s, 1H), 4.77 (t, J = 5.2 Hz, 1H), 4.46 (m, 1H), 4.30 (m, 1H), 4.19 (ttt, J = 8.4, 5.6, 2.5 Hz, 2H), 1.69 (s, 3H). ^{31}P NMR (162MHz, D_2O) δ -7.16. ^{13}C NMR (100MHz, D_2O) δ 164.73 (d, *J* = 8.7 Hz), 150.85, 148.51, 146.02, 142.06, 135.28 (d, *J* = 6.6 Hz), 128.86, 118.61, 87.97, 83.96 (d, *J* = 8.5 Hz), 74.04, 70.28, 65.65 (d, *J* = 5.9 Hz), 16.99. $[M+H]^+$ *m/z* calculated 416.0966 (100%), 417.0999 (15%), observed 416.0961 (100%), 417.0987 (10%).

Synthesis of Intermediate 2

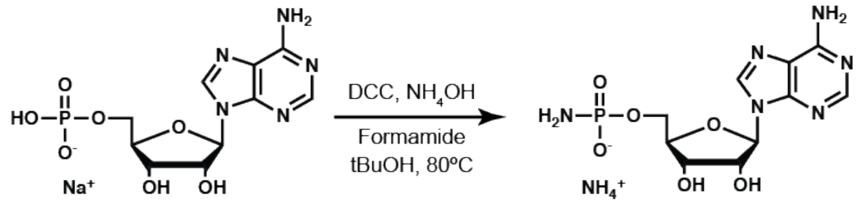

2

((2*R*,3*S*,4*R*,5*R*)-5-(6-amino-9*H*-purin-9-yl)-3,4-dihydroxytetrahydrofuran-2-yl)methyl hydrogen phosphoramidate (intermediate 2). The protocol for the synthesis of this intermediate was previously described (2). Adenosine 5'-monophosphate sodium salt (Sigma, 1041.7mg, 3.0mmol) was dissolved in ammonium hydroxide (2N, 7.5mL) and formamide (5mL). To this solution, a suspension of N,N-dicyclohexylcarbodiimide (DCC, 3095mg, 15.0mmol, 5eq) in tert-butanol (20mL) was added and the resulting two-phase reaction was heated to 80ºC. After 2-3hr, the solution becomes homogenous and after an additional 7hrs, the reaction is allowed to cool overnight. Unreacted DCC was removed by filtration and washed with water and the resulting solution was evaporated under reduced pressure to remove volatiles. The resulting solution was extracted with diethyl ether, then the aqueous layer was evaporated to dryness. Acetone was added to the resulting oil and the ammonium salt of the product was isolated by filtration (white gummy solid; 884.3mg, 81.8% yield).

Synthesis of AMP-acrylamide

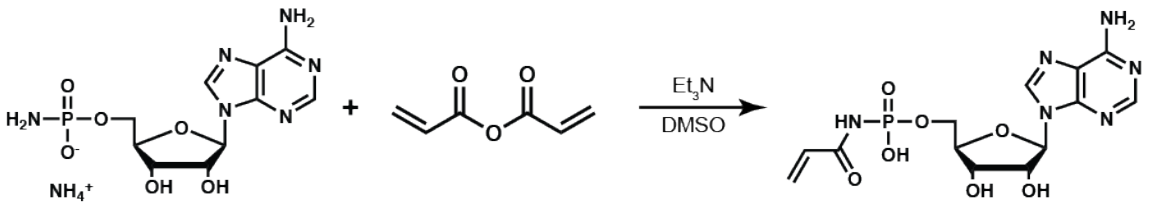

2 1 **AMP-acrylamide**

((2*R*,3*S*,4*R*,5*R*)-5-(6-amino-9*H*-purin-9-yl)-3,4-dihydroxytetrahydrofuran-2-yl)methyl hydrogen acryloylphosphoramidate (AMP-acrylamide). In a 4mL intermediate 2 (50mg, 0.138mmol) was dissolved in DMSO (1mL) and triethylamine (48µL, 0.344mmol, 2.5eq) with sonication and stirring. In a separate vial, intermediate 1 (19.1mg, 0.151mmol, 1.1eq) was dissolved in DMSO (100µL) and added slowly to the reaction vial. After 30min, the reaction was quenched by addition of 1% formic acid (1mL) and purified by reverse phase HPLC (0-30% acetonitrile/water) to yield the pure compound (white solid, 4.93mg, 8.5% yield). ^{1}H NMR (400MHz, D_2O) δ 8.46 (s, 1H), 8.24 (s, 1H), 6.09 (d, J = 1.7 Hz, 1H), 6.05 (d, J = 5.4 Hz, 1H), 5.65 (d, J = 10.4 Hz, 1H), 4.73 (d, J = 5.3 Hz, 1H), 4.44-4.37 (m, 1H), 4.29 (d, J = 2.6 Hz, 1H), 4.09 (tddt, J = 11.7, 9.3, 5.8, 3.0 Hz, 2H). ^{31}P NMR (162MHz, D_2O) δ -4.69. ^{13}C NMR (100MHz, D_2O) δ 169.48, 153.02, 149.14, 148.70, 141.09, 129.98, 129.20, 118.59, 87.52, 83.91 (d, *J* = 9.0 Hz), 74.19, 70.37, 64.83 (d, *J* = 5.3 Hz). $[M+H]^+$ *m/z* calculated 401.0969 (100%), 402.1003 (14%), observed 401.0957 (100%), 402.1006 (11%).

Synthesis of Adefovir-acrylate

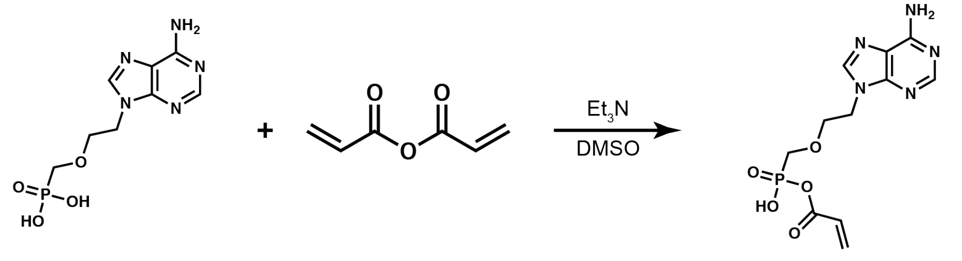

1 **Adefovir-acrylate**

acrylic ((2-(6-amino-9*H*-purin-9-yl)ethoxy)methyl)phosphonic anhydride (Adefovir-acrylate). In a 4mL scintillation vial, 9-[2-(Phosphonomethoxy)ethyl]adenine (PMEA, Adefovir) (AK Scientific, 20mg, 0.0732mmol) was dissolved in DMSO (1mL) and triethylamine (102µL,0.732mmol, 10eq) with sonication and stirring. In a separate vial, intermediate 1 (10.2mg, 0.0805mmol, 1.1eq) was dissolved in DMSO (100µL) and added slowly to the reaction vial. After 2hr, the reaction was quenched by addition of 1% formic acid (1mL) and purified by reverse phase HPLC (0-25% acetonitrile/water) to yield the pure compound (white solid, 18.4mg, 72% yield). ^{1}H NMR (400MHz, DMSO-d6 + Et_3N) δ 8.16 (s, 1H), 8.12 (s, 1H), 7.16 (s, 2H), 6.21 (dd, *J* = 17.2, 1.9 Hz, 1H), 6.12-6.04 (m, 1H), 5.86 (dd, *J* = 10.1, 1.9 Hz, 1H), 4.27 (t, *J* = 5.2 Hz, 2H), 3.85 (t, *J* = 5.2 Hz, 2H), 3.60 (d, *J* = 8.1 Hz, 2H). ^{31}P NMR (162MHz, DMSO-d6 + Et_3N) δ 9.67 (t, *J* = 8.0 Hz). ^{13}C NMR (100MHz, DMSO-d6 + Et_3N) δ 163.24 (d, *J* = 7.8 Hz), 156.35, 152.73, 149.92, 141.66, 131.84, 130.66 (d, *J* = 3.7 Hz), 118.97, 70.09 (d, *J* = 9.2 Hz), 68.36 (d, *J* = 154.7 Hz), 43.01. $[M+H]^+$ *m/z* calculated 328.0805 (100%), 329.0839 (12%), observed 328.0799 (100%), 329.0854 (13%).

SUPPLEMENTARY REFERENCES

1. Hilliker, A., Gao, Z., Jankowsky, E., and Parker, R. (2011) The DEAD-box protein Ded1 modulates translation by the formation and resolution of an eIF4F-mRNA complex. *Mol. Cell*. **43**, 962–972
2. Chambers, R. W., and Moffatt, J. G. (1958) The synthesis of adenosine-5“ and uridine-5” phosphoramidates. *J. Am. Chem. Soc.* **80**, 3752–3756
